# Editorial: Molecular and cellular mechanisms of heart failure: pathophysiology, pathogenesis and therapeutics

**DOI:** 10.3389/fcvm.2023.1260483

**Published:** 2023-08-04

**Authors:** Sean Lal, Kenneth Campbell, Amy Li

**Affiliations:** ^1^ School of Medical Sciences, The University of Sydney, Camperdown, NSW, Australia; ^2^ Department of Physiology, University of Kentucky, Lexington, KY, United States; ^3^ Department of Rural Clinical Sciences, La Trobe Rural Health School, La Trobe University, Flora Hill, VIC, Australia; ^4^ Centre for Healthy Futures, Torrens University Australia, Surrey Hill, NSW, Australia

**Keywords:** heart failure, cardiomyopathy, molecular, cellular, pathophisiology

**Editorial on the Research Topic** Molecular and cellular mechanisms of heart failure: pathophysiology, pathogenesis and therapeutics

Heart failure is the common endpoint of a diverse group of cardiac disorders including inherited or acquired (or a combination of the two) cardiomyopathies, myocardial ischemia and pressure/volume overload from valvular disease. Despite continuous advances in therapeutics, heart failure remains a major burden on the health care system with a high incidence of morbidity and mortality (up to 75% at 5 years) (1). Several physiological processes contribute to the development of heart failure including impaired contractility, alterations in energy metabolism, neurohormonal dysregulation, and maladaptive remodeling of the myocardium (2). Within cardiomyocytes, aberrant calcium handling and modulation of contractile proteins and ion channels caused by genetic mutations or post-translational modification are key contributors to impaired contractility. The collection of research papers in this issue highlights recent advances at the molecular and cellular level that contribute to the pathophysiology of heart failure with a major focus on cardiomyopathies, followed by insights gained from chronic multi-systemic diseases. Collectively, the mechanistic insight revealed by the authors offer novel therapeutic approaches and/or targets that are gaining traction towards translation in patients.

Molecular studies in this issue provide valuable insights into the pathogenesis of heart failure. Suppression of lusitropy is recognized as a contributor to impaired relaxation and diastolic dysfunction, a key mechanism in cardiomyopathies (Marston and Pinto). Marston and Pinto discuss how altered calcium handling by protein kinase A (PKA) phosphorylation of troponin I and how phospholamban impacts lusitropy. They identify therapeutic targets in conditions where diastolic dysfunction is prevalent. Similarly, S-glutathionylation of sarcomere proteins has emerged as a molecular modifier in contractile dysfunction and disease progression associated with oxidative stress (Rosas and Solaro). In this study, Rosas and Solaro outline the possibility of screening serum S-glutathionylated sarcomeric proteins as a clinical tool for early diagnosis in patients at risk of heart failure, stratification and determination of progression, and even to identify the effectiveness of new therapeutic approaches. Another way to investigate the functional impact of specific phosphorylation sites is to engineer amino acid substitutions that mimic phosphorylation, known as phosphomimetics. This was accomplished by Kazmierczak et al. who demonstrated the therapeutic potential of myosin regulatory light chain phosphomimetics by showing that they could rescue the contractile defects caused by hypertrophic cardiomyopathy.

Besides post-translational modification, dysfunctional cytoskeletal and calcium cycling regulators have been implicated in atrial and ventricular dysfunction and susceptibility to arrhythmias (Grogan et al.) (3). Grogan et al. examined the functional deletions of the Obscurin immunoglobulin 58/59 domains (Ig58/59) and demonstrated their essential role in regulating cytoskeleton structure and Ca^2+^ cycling in the atria. This new knowledge provides a mechanistic explanation of the previously characterized phenotype with an onset of atrial enlargement and fibrillation in the ageing heart (4). Additionally, Dewi et al. discovered a genetic susceptibility of peripartum cardiomyopathy by linking the association of guanine nucleotide–binding protein β3 subunit (GNB3) C825T polymorphism and insertion/deletion of the angiotensin-converting enzyme (ACE) gene to the disease. Unravelling these molecular mechanisms and their functional consequences opens new avenues for therapeutic interventions aimed at restoring normal cardiac function.

The next set of studies illustrate the cellular processes that contributes to heart failure pathogenesis. Pun et al. provide a comprehensive review of Connexin 43, an essential gap junction protein localized to the intercalated discs which connect adjacent cardiomyocytes. Phosphorylation of connexin 43 S368 by protein kinase C (PKC) has been identified as a key regulator of cardiac function where both hypo- and hyper-phosphorylation are associated with disease, impacting cell-cell coupling and arrhythmogenesis (Pun et al.). Transcriptional burst was an interesting concept first described by the Kraft group in hypertrophic cardiomyopathy patients with heterogenous mutations (5). This causes independent transcription of mutated and wild-type alleles giving rise to a mosaic expression in the ratio of mRNA between individual cardiomyocytes. Burkart et al. demonstrated that sarcomeric proteins also exhibited mosaic patterns and contributed to highly variable Ca^2+^-dependent force generation between individual cardiomyocytes. The contractile imbalance between individual cardiomyocytes promotes HCM-development contributing to disease progression (Burkart et al.).

Three further articles in this issue consider the role of adverse cardiac remodeling and fibrosis induced by myofibroblasts in Flinn et al., chronic kidney disease in Kishimoto et al., and chronic intermittent hypoxia in Xuan et al. These diverse pathological pathways underpin the cardiac consequences of many multi-systemic diseases.

Advancements in understanding of heart failure pathophysiology have paved the way for innovative therapeutic approaches targeting specific molecular and cellular mechanisms. Novel tools such as affimers, extracellular vesicles and long non-coding RNAs (lncRNAs) offer new possibilities for diagnostic and therapeutic development. Parker et al. provide evidence that affimers, small engineered non-antibody binding proteins, can target proteins in cardiomyocytes. This proof-of-concept offers a novel tool that improves imaging of heart tissue, aiding in diagnosis and monitoring of heart failure (Parker et al.). New factors, such as the shedding of extracellular vesicles by adaptive and innate immune cells confirmed by Vilella-Figuerola et al., unveil previously unrecognized contributors to heart failure pathophysiology. Moreover, long non-coding RNAs (lncRNAs) have emerged as an important epigenetic regulator of DNA, RNA and proteins with diverse implications in heart failure pathogenesis (6). Fan et al. conducted a comprehensive literature review of lncRNAs’ involvement in heart failure that highlights their potential as diagnostic markers and epigenetic-based therapeutic targets. These advances provide opportunities to developing personalized therapeutic strategies beyond the now standard therapeutic inhibitors and highlight the increasing interaction between genetic pre-disposition and environment.

This topical issue represents some of the most recent progress in our understanding of the complex cellular mechanisms underlying heart failure. The suppression of lusitropy, S-glutathionylation of sarcomere proteins, connexin 43 phosphorylation, alterations in cytoskeletal and calcium cycling regulators, and transcriptional heterogeneity in cardiomyocyte are some of the key molecular mechanisms implicated in cardiomyopathies. Insights into cellular processes, such as myofibroblast regulation, signaling pathways, and the impact of chronic intermittent hypoxia provide valuable information underpinning the more generalized progression towards heart failure. Together, the research presented in this issue opens new avenues for the development of targeted diagnostics and design of novel treatment strategies that improve cardiac function, prevent aberrant myocardial remodeling, and reduce fibrosis. These measures could be aimed at restoring normal cardiac function and ultimately improve patient outcomes.
